# *BRCA1* promoter hypermethylation in sporadic epithelial ovarian carcinoma: Association with low expression of BRCA1, improved survival and co-expression of DNA methyltransferases

**DOI:** 10.3892/ol.2014.1878

**Published:** 2014-02-13

**Authors:** XUEFENG BAI, YINGZI FU, HUI XUE, KEJUN GUO, ZHIGUO SONG, ZHAOJIN YU, TIANHONG JIA, YUANYUAN YAN, LIN ZHAO, XIAOYI MI, ENHUA WANG, ZHIHONG ZHENG, HAISHAN ZHAO, WEIFAN YAO, MINJIE WEI

**Affiliations:** 1 Department of Pharmacology, School of Pharmacy, China Medical University, Shenyang, Liaoning 110001, P.R. China; 2 Department of Obstetrics and Gynecology, First Affiliated Hospital, China Medical University, Shenyang, Liaoning 110001, P.R. China; 3 Department of Pathology, College of Basic Medical Sciences, China Medical University, Shenyang, Liaoning 110001, P.R. China

**Keywords:** ovarian cancer, breast cancer susceptibility gene 1, methylation, DNA methyltransferases, immunohistochemistry

## Abstract

The breast cancer susceptibility gene 1 (*BRCA1*) inactivation in sporadic epithelial ovarian carcinoma (EOC) is common and low BRCA1 expression is associated with promoter hypermethylation. The clinical validation of *BRCA1* methylation as a prognostic marker in EOC remains unresolved. The aim of the present study was to determine the aberrant promoter methylation of *BRCA1* in benign and malignant ovarian tumor tissues, to establish the association with the clinicopathological significance and the prognostic value. Additionally, the contribution of DNA methyltransferase (DNMT) expression to *BRCA1* promoter hypermethylation was determined. The rate of *BRCA1* methylation was observed to be 35.2% (50/142) in the EOCs; however, no methylation (0/32) was observed in the benign tumors. *BRCA1* methylation was significantly associated with the downregulation of *BRCA1* expression (P<0.001) and the frequency of *BRCA1* methylation was greater in the carcinomas of patients whose tumor was bilateral than that of patients with a unilateral carcinoma (P=0.015). *BRCA1* methylation was significantly associated with the preoperative serum carbohydrate antigen-125 level (P=0.013), improved overall survival (P=0.005) and disease-free survival (P=0.007). In addition, a significant correlation was observed between the co-expression of DNMTs and the methylation status of *BRCA1*. Thus, the present study provided support for *BRCA1* promoter hypermethylation as a prognostic marker for survival in sporadic EOC, and co-expression of DNMTs was observed to contribute to *BRCA1* promoter hypermethylation.

## Introduction

Epithelial ovarian carcinoma (EOC) is a predominant, lethal gynecologic malignancy, with a five-year survival rate of <25% for patients that are diagnosed with stage III–IV disease (1,2). The treatment for EOC remains challenging, regardless of advances in surgical procedures and chemotherapy. The current ovarian cancer classification scheme distinguishes tumors based on the histopathological subtype, grade and surgical stage. Although established differences are observed in the clinical behavior, all of the ovarian neoplasia are subject to the same treatment paradigm. Therefore, the development of predictive markers that will direct the treatment selection, in addition to novel targeted therapies, are required for the treatment of EOC.

Aberrant DNA methylation is currently recognized as a common molecular abnormality in cancer. The abnormal promoter methylation may be a potential molecular marker for cancer diagnosis, prognosis and treatment (3–5). Recent studies have focused on the identification of methylated genes and the evaluation of their potential application as biomarkers for the prognosis and diagnosis of EOC (6,7). However, it is particularly unclear which genes should be used to identify a methylator phenotype that may become instrumental in the clinical diagnosis, prognosis and treatment of cancer.

The breast cancer susceptibility gene 1 (*BRCA1*) is a significant breast and ovarian cancer susceptibility gene; it has been mapped to chromosome 17q21 and encodes a nuclear protein, which is comprised of 1863 amino acids (8). *BRCA1* contributes to the regulation of transcriptional activation, DNA repair, apoptosis, cell-cycle checkpoint control and chromosomal remodeling (9,10). BRCA1 dysfunction, frequently observed in high-grade serous ovarian carcinomas, commonly results from germline and somatic mutations, as well as promoter methylation. Therefore, the identification of tumors exhibiting BRCA1 defects has therapeutic and prognostic implications (11–16). The hypermethylation of the *BRCA1* promoter is important in silencing *BRCA1* in sporadic EOCs, and previous studies have demonstrated that methylation of *BRCA1* is associated with chemotherapy sensitivity and patient survival (17–20). *BRCA1* CpG island hypermethylation predits sensitivity to poly (ADP-ribose) polymerase-1 inhibitors (21), thus indicating a potential role for *BRCA1* methylation as a biological marker in the clinical treatment of EOC.

DNA methyltransferases (DNMTs) are a family of enzymes responsible for the transfer of methyl groups to cytosine (22,23) and the DNMT family includes DNMT1, 2, 3a, 3b and 3L (24,25). DNMT2 (whose substrate and DNA methylation activity is unclear) (26), was shown to methylate transfer RNA (27,28) and DNMT3L (which is essential for establishing maternal genomic imprints, however, lacks key methyltransferase motifs) is potentially a regulator of methylation rather than an enzyme that methylates DNA (29). It has been suggested that that global genomic DNA methylation patterns are established and predominantly maintained by the combined action of three enzymatically active DNMTs, 1, 3a and 3b. DNMT1 is traditionally referred to as a maintenance enzyme as it copies methylation following replication, whereas DNMT3a and DNMT3b are *de novo* enzymes, which establish novel patterns of methylation during differentiation (30,31). Aberrant expression of DNMTs and disruption of DNA methylation patterns are closely associated with numerous types of cancer. Our previous study demonstrated that DNMT3a expression was higher in EOC (32), indicating an important role for the differential expression of DNMTs in aberrant DNA methylation patterns of EOC. However, there is currently no data concerning the association between DNMT expression and DNA methylation in EOC.

In the present study, the expression and methylation of *BRCA1* was investigated in the same cohort of ovarian tumor patients as our previous study (32) and the association between BRCA1 expression and methylation was analyzed. The clinicopathological and prognostic differences, based on *BRCA1* promoter methylation in EOC patients, were analyzed to determine the role of *BRCA1* methylation in EOC and to understand the clinical significance. Furthermore, the association between *BRCA1* methylation and DNMT protein expression was analyzed to investigate the role of DNMTs in *BRCA1* methylation.

## Patients and methods

### Patients and tissue samples

The present study included 142 EOC cases and 32 ovarian benign tumor cases, which were selected from the Department of Surgical Oncology and General Surgery at the China Medical University-Affiliated First and Second Hospitals (Liaoning, China). All of the cases included in the present study were clinical cases that were routinely examined and diagnosed between 2002 and 2010. None of the patients had a family history of cancer and they were surgically staged according to the current International Federation of Gynecologists and Obstetricians (FIGO) classification system. The histological diagnosis was determined based on criteria set out by the World Health Organization (33). Approval for the present study was obtained from the Institute Research Medical Ethics Committee of the China Medical University (Liaoning, China). Consent was obtained from the families of all patients.

### Immunohistochemical analysis

Formalin-fixed and paraffin-embedded tissue samples were cut into 4-μm sections and mounted onto poly-L-lysine-coated glass slides. For immunohistochemical staining, the sections were deparaffinized in xylene, rehydrated in a series of alcohol and washed using tap water. The sections were autoclaved in 10 mM sodium citrate buffer (pH 6.0; China National Pharmaceutical Group Corporation, Beijing, China) for 10 min for antigen retrieval. Endogenous peroxidase activity was blocked by incubating the sections in 3% H_2_O_2_ (China National Pharmaceutical Group Corporation) at 37°C for 20 min. The sections were blocked for non-specific binding by 10% normal goat serum at 37°C for 30 min and were incubated at 4°C overnight with mouse monoclonal anti-*BRCA1* antibodies (1:100; MS110, Calbiochem, Darmstadt, Germany). The following day, the sections were washed three times with 0.01 mol/l phosphate-buffered saline (PBS; pH 7.4) for 15 min and incubated with a goat anti-mouse IgG secondary antibody (catalogue no. sc-2039; Santa Cruz Biotechnology Inc., Santa Cruz, CA, USA) for 30 min at 37°C. Subsequently, the sections were incubated with a streptavidin horseradish peroxidase solution (LSAB^TM^ kit, Dako, Glostrup, Denmark) for 30 min, washed with PBS and stained with 3,3′-diaminobenzidine. The sections were then counterstained with Mayer’s hematoxylin (Sigma-Aldrich, St. Louis, MO, USA), dehydrated and mounted. The negative controls were generated using PBS as a replacement for the anti-BRCA1 antibodies.

### Evaluation of immunohistochemistry

The immunostained sections were independently reviewed and scored by two investigators who were blinded to the clinicopathological characteristics of the patients; a positive correlation was observed between them. The nuclear positivity of the BRCA1 proteins was evaluated using semi-quantitative scoring criteria according to the staining intensity (0, negative; 1, weak; 2, moderate; and 3, strong) and the proportion of positive cells (0, negative; 1, positive in ≤10%; 2, positive in >10% and ≤50%; 3, positive in >50% and ≤80%; 4, positive in >80% of tumor cells). The two scores were multiplied together for each case and the expression was graded as: 0, negative score; 1–4, weak expression score; 5–8, moderate expression score; and 9–12, strong expression score.

### Methylation-specific polymerase chain reaction (MSP)

The promoter methylation status of the *BRCA1* gene was analyzed using MSP. The bisulfite modification, primer sequences and PCR conditions were performed as previously described (29). Lymphocyte DNA, which was treated with *Sss*I bacterial methylase (New England BioLabs, Hithcin, UK) served as the positive control for the methylated alleles and the DNA from normal lymphocytes served as the control for the unmethylated alleles; the negative controls, without DNA, were included in each experiment. All PCR reactions were conducted in duplicate and a methylated band, which was detected in either or both duplicates, was recorded as positive for promoter methylation.

### Statistical analysis

Pairwise correlations between the categorical variables were investigated using the χ^2^ test or Fisher’s exact test where appropriate. Overall survival (OS) and disease-free survival (DFS) were estimated using the Kaplan-Meier method, and the log-rank test was used to compare the patient survival time between or among the groups. The Cox proportional hazards model (Cox regression) was used with backward elimination to identify the significant, independent, prognostic factors. OS was defined as the time interval from initial surgery to mortality or, for surviving patients, the time interval between the initial surgery and the final follow-up. DFS was defined as the time interval between the initial surgery and the date of disease progression or recurrence, or the final follow-up. The statistical tests were two-sided and P<0.05 was considered to indicate a statistically significant difference. All statistical analyses were performed using the SPSS statistical software program (SPSS, Chicago, IL, USA).

## Results

### Patient characteristics

In the present study, the tissue sections were obtained from 174 ovarian tumor samples for the evaluation of BRCA1 protein expression and promoter methylation. The clinicopathological data from the patients are shown in Table I. Briefly, the mean age of the patients at the time of surgery was 53 years (range, 20–74 years). Twenty-seven (23.3%) patients exhibited lymph node-metastasized disease at the time of surgery and 110 (80.9%) patients exhibited serous carcinoma as the predominant histological diagnosis, followed by mucinous carcinoma (8.5%), clear cell carcinoma (5.6%) and undifferentiated carcinoma (5.6%). The preoperative serum levels of carbohydrate antigen (CA)-125 and CA19-9 were elevated prior to surgery (higher than in the levels of healthy individuals) in the majority of patients but none of the patients received any neo-adjuvant chemotherapy. Follow-up data were available for 85 patients. The mean and median OS times were 56.1 and 41.0 months with 95% confidence intervals (CIs) of 45.3–66.9 and 33.2–48.8 months, respectively. The mean and median DFS times were 46.6 and 26.0 months with 95% CIs of 36.4–56.9 and 14.6–37.4 months, respectively. The 32 benign tumors comprised of 27 that were serous and five that were mucinous.

### BRCA1 promoter methylation is associated with low BRCA1 expression in EOC

The methylation of *BRCA1* was detected in 142 cases of malignant tumors and 32 cases of benign, ovarian tumors. *BRCA1* methylation was detected in 50 (35.2%) of the 142 carcinomas, however, no methylation was detected in the benign tumors. The frequency of *BRCA1* methylation was significantly higher in the EOC samples than that observed in the benign tumor samples (P<0.001, Pearson’s χ^2^ test).

The BRCA1 expression in the 142 carcinoma samples was as follows, 67 (47.2%) were negative and 62 (43.7%) were weakly positive, nine (6.3%) were moderately positive and four (2.8%) were strongly positive for BRCA1. Among the 32 cases of benign tumors; nine (28.1%) were negative and 15 (46.9%) were weakly positive, four (12.5%) were moderately positive and four (12.5%) were strongly positive for nuclear BRCA1. The expression of BRCA1 in the carcinoma samples was significantly reduced compared with that observed in the benign tumors (P=0.031, Pearson’s χ^2^ test).

**Figure 1:**
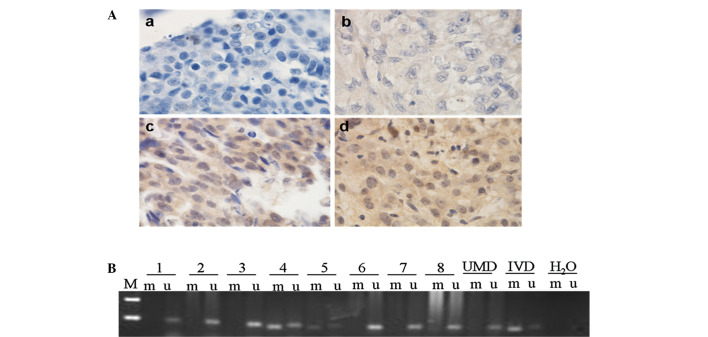
Immunohistochemical staining and methylation of *BRCA1* in sporadic EOC tissues. (A) Immunohistochemical staining of sporadic EOC specimens (magnification, ×1000): (a) Negative stain; (b) weak positive stain; (c) moderately positive stain; (d) strongly positive stain. (B) Methylation of *BRCA1*. MSP was performed on bisulfite-treated DNA from ovarian cancer cells. MSP results from nine representative patients are shown. The DNA bands in the u-labeled lanes indicate PCR products that were amplified with primers recognizing the unmethylated promoter sequence. The DNA bands in m-labeled lanes represent the products that were amplified with methylation-specific primers. The DNA from the normal lymphocytes served as the control for UMD and IVD served as the control for methylated DNA. H_2_O was used as a template for the negative control. M, marker; UMD, unmethylated DNA; IVD, *in vitro* methylated DNA; *BRCA1*, breast cancer susceptibility gene 1; EOC, epithelial ovarian carcinoma; MSP, methylation-specific polymerase chain reaction.

In the EOC samples, a reduction of the BRCA1 protein was significantly associated with *BRCA1* methylation (P<0.001, Pearson’s χ^2^ test; Table II). Representative results of the immunohistochemical staining and *BRCA1* promoter methylation in EOC tissues are shown in Fig. 1.

### BRCA1 promoter methylation is associated with clinicopathological parameters in EOC

The association between *BRCA1* promoter methylation and clinicopathological parameters is shown in Table III. The data indicated that *BRCA1* methylation was significantly associated with tumor localization. In addition, the frequency of *BRCA1* methylation was observed to be higher in the patients whose tumor was bilateral than that of patients whose tumor unilateral (P=0.015, Pearson’s χ^2^ test). Moreover, the methylation of the *BRCA1* gene was marginally associated with the FIGO clinical stage (P=0.085, Pearson’s χ^2^ test).

CA-125, CA19-9 and carcinoembryonic antigen (CEA) are the predominant biomarkers for confirming the diagnosis and management of EOC. Although they commonly aid with the diagnosis of ovarian malignancies, there are significant limitations relating to their sensitivity and specificity. The association between *BRCA1* gene promoter methylation and preoperative serum levels of CA-125, CA19-9 and CEA were analyzed in the present study and *BRCA1* methylation was observed to be significantly associated with the CA-125 level. The frequency of *BRCA1* methylation was greater in patients exhibiting higher preoperative CA-125 levels than that observed in patients exhibiting lower CA-125 levels (P=0.013, Pearson’s χ^2^ test).

### BRCA1 promoter methylation is associated with an improved survival rate

**Figure 2:**
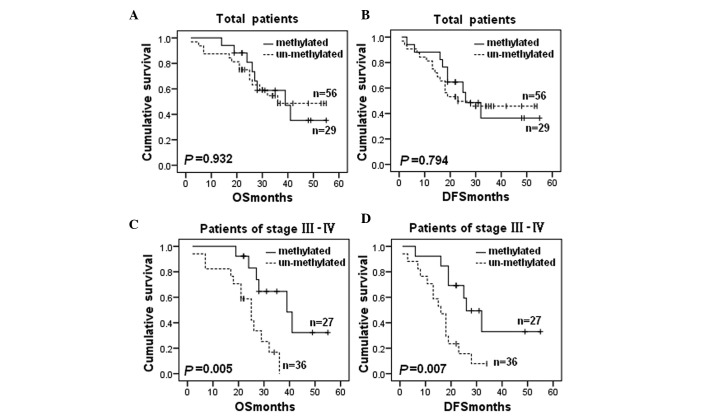
Kaplan-Meier curves for OS and DFS according to the *BRCA1* methylation status. (A) *BRCA1* methylation and OS in all cases (P=0.932). (B) *BRCA1* methylation and DFS in all cases (P=0.794). (C) *BRCA1* methylation and OS in the subgroups of stage III–IV cases (P=0.005). (D) *BRCA1* methylation and DFS in the subgroups of stage III–IV cases (P=0.007). P-value obtained from log-rank test. OS, overall survival; DFS, disease-free survival; *BRCA1*, breast cancer susceptibility gene 1.

In the present study *BRCA1* methylation did not correlate with the patient survival rate in all of the patients (OS: P=0.932; Fig. 2A and DFS: P=0.794; Fig. 2B). However, in the patients exhibiting advanced stage (III–IV) cancer, *BRCA1* methylation was significantly associated with improved OS (P=0.005; Fig. 2C) and DFS (P=0.007; Fig. 2D).

Cox regression univariate analysis of the potential prognostic impact of the clinical and histopathological parameters enabled identification of the FIGO clinical stage (OS: P=0.003; RR=8.750; 95% CI, 2.072–36.951; and DFS: P=0.001; RR=7.446; 95% CI, 2.279–24.331), the location of the tumor (OS: P=0.075; RR=2.019; 95% CI, 0.931–4.376; and DFS: P=0.024; RR=2.227; 95% CI, 1.112–4.462) and the serum CA-125 level (OS: P=0.113; RR=1.688; 95% CI, 0.884–3.224; and DFS, P=0.007; RR=2.067; 95% CI, 1.218–3.508) are significantly associated with a reduced OS and DFS (Table IV). Subsequently, multivariate Cox regression models using the clinical stage, tumor size, location of neoplasia and *BRCA1* methylation revealed that the clinical stage alone provided the independent prognostic factor (OS: P=0.012; RR=7.315; 95% CI, 1.552–34.481; and DFS: P=0.008; RR=5.535; 95% CI, 1.552–19.739) (Table V).

### DNMT co-expression is associated with BRCA1 methylation in EOC

In the present study the correlation between DNMT expression and *BRCA1* methylation was analyzed (Table VI). No significant correlation between the methylation status of *BRCA1* and DNMT1, 3a, or 3b protein expression was observed (P=0.135, P=0.824 and P=0.260, respectively). However, a significant correlation was observed between the methylation status of *BRCA1* and the co-expression of any two of the DNMTs or all three of the DNMTs. The methylation rates of *BRCA1* in the samples exhibiting co-expression of DNMT1 and 3a, DNMT1 and 3b, or DNMT3a and 3b, were greater than the rates observed in the non-co-expression samples (P=0.030, P=0.034 and P=0.046, respectively). Additionally, when all three of the DNMTs were positive, the highest *BRCA1* methylation rate was observed (59.0%).

## Discussion

The loss of expression of tumor suppressor genes is known to occur via biallelic inactivation (35). The current consensus is that the somatic mutation of *BRCA1* is rare in sporadic EOC, thus, the loss of BRCA1 expression is considered to be due to a combination of allelic loss and methylation or biallelic methylation (36,37). Promoter methylation is significant in silencing *BRCA1*. The varying experimental methods and, in particular, the region of the *BRCA1* promoter that has been investigated across studies have identified a range of frequencies of *BRCA1* promoter methylation, from 5 to 40% of clinical specimens (38). In the present study, the methylation rate of *BRCA1* in EOC samples is consistent with that observed in previous studies and *BRCA1* promoter methylation was significantly associated with a decreased expression of *BRCA1*. These results corroborated the hypothesis that *BRCA1* hypermethylation was a predominant cause of BRCA1 loss of expression.

Previous studies regarding the association between *BRCA1* gene promoter methylation and patient survival remain controversial. Montavon *et al* (6) and Yang *et al* (39) indicated that *BRCA1* methylation was not associated with patient survival, however, another study demonstrated a survival disadvantage in patients whose neoplasms were methylated at *BRCA1* (17). In the present study, a significant association was observed between *BRCA1* methylation and an improved survival rate in patients with an advanced FIGO stage (III–IV). The inconsistency of these results may be due to the varying regions of the *BRCA1* promoter that were investigated in addition to the different populations that were involved in the studies. The data from the present study resulted in the hypothesis that the cells from the patients with methylation of the *BRCA1* promoter may exhibit an impaired ability to repair DNA damage via downregulation of *BRCA1* expression. The patients may experience an increased sensitivity to platinum chemotherapy, resulting in an improved survival outcome. These results were consistent with previous studies, which demonstrated that *BRCA1* gene promoter methylation may be an effective indicator of the patient response to chemotherapy (20,21). As there were limitations in the present study due to the small number of cases, further investigation is required to clarify the role of *BRCA1* gene methylation in chemoresponsiveness and patient survival in EOC cases.

The present study indicated that *BRCA1* methylation was significantly associated with the tumor location. Furthermore, the frequency of *BRCA1* methylation was observed to be greater in patients with bilateral ovarian cancer than in unilateral cancer patients, which may indicate a contrast between the unilateral and bilateral ovarian cancers regarding their biological characteristics, genetics and mechanisms of carcinogenesis. Moreover, these data indicated that *BRCA1* methylation was significantly associated with the preoperative serum CA-125 levels. CA-125 was established as a prognostic marker in cancer, specifically in ovarian carcinoma (40) and the present results indicated that *BRCA1* methylation and CA-125 levels, in combination, may be used as biomarkers for the diagnosis and prognosis of EOC.

The causes of DNA methylation at specific CpG islands in cancer are ambiguous and potentially multifactorial. DNMTs were a predominant cause of DNA methylation and the differential expression of DNMTs has been identified in numerous types of cancer (41–43), including EOC, which we previously reported (32). It was hypothesized in the present study that the over-expression of DNMT proteins may contribute to *BRCA1* methylation; therefore, the association between *BRCA1* methylation and DNMT protein expression was analyzed. The results indicated that there was no significant correlation identified between the methylation status of *BRCA1* and the individual protein expression of DNMT1, 3a or 3b. However, the co-expression of DNMTs was identified to be significantly associated with *BRCA1* methylation. The results of the present study supported the hypothesis that genomic methylation patterns may be established depending on the interaction of these three enzymes. Further studies are required to clarify how these DNMTs interact with each other.

In conclusion, *BRCA1* gene promoter methylation was identified to be significant in reducing protein expression in a Chinese population and the co-expression of DNMTs may have contributed to *BRCA1* gene promoter hypermethylation. Moreover, *BRCA1* gene promoter hypermethylation was observed to be significantly associated with a favorable survival rate, thus supporting the hypothesis that *BRCA1* methylation may be an important prognostic marker for EOC.

## Figures and Tables

**Table I:** Patient characteristics.

Characteristic	n	%
Age (years)		
≤53	79	57.2
>53	59	42.8
Unknown	4	-
Menopause state		
Pre-menopause	45	34.6
Post-menopause	85	65.4
Unknown	12	-
Histological type		
Serous	110	77.5
Mucinous	12	8.5
Clear cell	8	5.6
Transitional	2	1.4
Endometrioid	2	1.4
Undifferentiated	8	5.6
Tumor size (cm)		
≤5	15	12.5
5–10	53	44.2
>10	51	43.3
Unknown	23	-
FIGO stage		
I–II	31	24.4
III–IV	96	75.6
Unknown	15	-
Node metastasis		
No	89	76.7
Yes	27	23.3
Unknown	26	-
Location of tumor		
Single side	56	43.4
Both sides	73	56.6
Unknown	13	-
CA-125 (U/ml)		
0–35	10	10.1
35–500	40	40.4
500–1000	32	32.3
>1000	17	17.2
Unknown	43	-
CA19–9 (U/ml)		
0–37	67	76.1
37–100	10	11.4
>100	11	12.5
Unknown	54	-
CEA (ng/ml)		
0–5	72	91.1
>5	7	8.9
Unknown	63	-
Chemotherapy		
Platinum-based	111	94.9
Non-platinum	3	2.6
No chemotherapy	3	2.6
Unknown	25	-

Unknown: these features were unavailable from the clinical-pathological records of the patients. FIGO, International Federation of Gynecology and Obstetrics; CA, carbohydrate antigen; CEA, carcinoembryonic antigen.

**Table II:** Correlation between *BRCA1* expression and *BRCA1* methylation in 142 sporadic EOC cases.

		*BRCA1* methylation, n (%)		
			
BRCA1 expression	n	Positive	Negative	P-value
Malignant	142	50 (35.2)	92 (64.8)	<0.001
Negative	67	35 (52.2)	32 (48.7)	
Positive	75	15 (20.0)	60 (80.0)	

*BRCA1*, breast cancer susceptibility gene 1; EOC, epithelial ovarian carcinoma.

**Table III:** Correlation between *BRCA1* promoter methylation and clinicopathological features of sporadic EOC patients.

		*BRCA1* methylation, n (%)		
			
Clinicopathological features	n	Positive [50 (35.2)]	Negative [92 (64.8)]	P-value^a^
Age at diagnosis (years)	138			
≤53	79	26 (32.9)	53 (67.1)	0.742
>53	59	21 (35.6)	38 (64.4)	
Menopause state	130			
Pre-menopause	45	13 (28.9)	32 (71.1)	0.318
Post-menopause	85	32 (37.6)	53 (62.4)	
Tumor size (cm)	119			
≤5.0	15	5 (33.3)	10 (66.7)	0.282
5–10	53	15 (28.3)	38 (71.7)	
>10	51	22 (43.1)	29 (56.9)	
Node metastasis	116			
No	89	31 (34.8)	58 (65.2)	0.834
Yes	27	10 (37.0)	17 (63.0)	
FIGO stage	127			
I–II	31	7 (22.6)	24 (77.4)	0.085
III–IV	96	38 (39.6)	58 (60.4)	
Histological type	130			
Serous	110	38 (34.5)	72 (65.5)	0.506
Mucinous	12	2 (16.7)	10 (83.3)	
Clear cell	8	3 (37.5)	5 (62.5)	
Tumor location	129			
Single side	56	11 (19.6)	45 (80.4)	0.015
Both sides	73	29 (39.7)	44 (60.3)	
CA-125 (U/ml)	89			
35–500	40	9 (22.5)	31 (77.5)	0.013
500–1000	32	18 (56.2)	14 (43.8)	
>1000	17	7 (41.2)	10 (58.8)	
CA19-9 (U/ml)	88			
0–37	67	24 (35.8)	43 (64.2)	0.083
>37	21	12 (57.1)	9 (42.9)	
CEA (U/ml)	79			
0–5	72	20 (27.8)	52 (72.2)	0.191
>5	7	4 (57.1)	3 (42.9)	

a Obtained from Pearson’s χ^2^ or Fisher’s exact test.

*BRCA1*, breast cancer susceptibility gene 1; EOC, epithelial ovarian carcinoma; FIGO, International Federation of Gynecology and Obstetrics; CA, carbohydrate antigen; CEA, carcinoembryonic antigen.

**Table IV:** Univariate Cox regression analysis of OS and DFS in EOC.

		OS			DFS		
			
Factor	n	RR	95% CI	P-value	RR	95% CI	P-value
Age (years)							
>53/≤53	85	1.283	0.638–2.583	0.485	1.330	0.724–2.442	0.357
Menopause state							
Post/pre-	83	1.187	0.532–2.649	0.676	1.335	0.668–2.667	0.413
Tumor size (cm)							
>5/≤5	70	1.599	0.371–6.889	0.529	1.759	0.418–7.399	0.441
Node metastasis							
Yes/no	68	1.695	0.708–4.055	0.236	1.114	0.483–2.571	0.799
Histological type							
Serous/non-serous	84	0.950	0.411–2.197	0.904	0.886	0.423–1.853	0.748
FIGO stage							
III–IV/I–II	79	8.750	2.072–36.951	0.003	7.446	2.279–24.331	0.001
Location of neoplasia							
Bilateral/unilateral	78	2.019	0.931–4.376	0.075	2.227	1.112–4.462	0.024
CA-125 (U/ml)							
>1000/500-1000/35-500	56	1.688	0.884–3.224	0.113	2.067	1.218–3.508	0.007
CA19-9 (U/ml)							
>37/0–37	53	0.366	0.085–1.579	0.178	0.399	0.119–1.342	0.138

OS, overall survival; DFS, disease-free survival; EOC, epithelial ovarian cancer; RR, relative risk; CI, confidence interval; FIGO, International Federation of Gynecology and Obstetrics; CA, carbohydrate antigen.

**Table V:** Multivariate Cox regression analysis of OS and DFS in EOC.

	OS		DFS	
		
Factor	RR (95% CI)	P-value	RR (95% CI)	P-value
Clinical stage				
III–IV/I–II	7.315 (1.552–34.481)	0.012	5.535 (1.552–19.739)	0.008
Tumor size (cm)				
>5/≤5	2.383 (0.534–10.636)	0.255	2.435 (0.567–10.458)	0.231
Location of neoplasia				
Bilateral/unilateral	1.210 (0.425–3.445)	0.721	1.429 (0.563–3.625)	0.453
*BRCA1* methylation				
Positive/negative	0.448 (0.157–1.281)	0.134	0.584 (0.256–1.332)	0.201

OS, overall survival; DFS, disease free survival; EOC, epithelial ovarian carcinoma; RR, relative risk; CI, confidence interval; *BRCA1*, breast cancer susceptibility gene 1.

**Table VI:** Correlation between DNMT expression and *BRCA1* methylation in 142 sporadic EOC cases.

		*BRCA1* methylation, n (%)		
			
Features	n	Positive [92 (64.8)]	Negative [50 (35.2)]	P-value^a^
DNMT3a status				
Negative	50 (35.2)	33 (66.0)	17 (34.0)	0.824
Positive	92 (64.8)	59 (64.1)	33 (35.9)	
DNMT3b status				
Negative	63 (44.4)	44 (69.8)	19 (30.2)	0.260
Positive	79 (55.6)	48 (60.8)	31 (39.2)	
DNMT1 status				
Negative	66 (46.5)	47 (71.2)	19 (28.8)	0.135
Positive	76 (53.5)	45 (59.2)	31 (40.8)	
DNMT1+DNMT3a				
No co-expression	88 (62.0)	63 (71.6)	25 (28.4)	0.030
Co-expression	54 (38.0)	29 (53.7)	25 (46.3)	
DNMT1+DNMT3b				
No co-expression	93 (65.5)	66 (71.0)	27 (29.0)	0.034
Co-expression	49 (34.5)	26 (53.1)	23 (46.9)	
DNMT3a+DNMT3b				
No co-expression	84 (59.2)	60 (71.4)	24 (28.6)	0.046
Co-expression	58 (40.8)	32 (55.2)	26 (44.8)	
DNMT1+DNMT3a+DNMT3b				
No co-expression	103 (72.5)	76 (73.8)	27 (26.2)	<0.001
Co-expression	39 (27.5)	16 (41.0)	23 (59.0)	

a Obtained from Pearson’s χ^2^ or Fisher’s exact test.

DNMT, DNA methyltransferase; *BRCA1*, breast cancer susceptibility gene 1.
